# BSA-Seq-Based QTL Mapping for the Height of the First Fruiting Branch Node of Cotton and the Development of Molecular Markers

**DOI:** 10.3390/genes17091030

**Published:** 2026-08-28

**Authors:** Fuxiang Zhao, Tao Yang, Xuwen Wang, Gang Wang, Jinxin Qiao, Xianhui Kong, Li Liu, Wanli Han, Yu Yu

**Affiliations:** 1 Cotton Research Institute, Xinjiang Academy of Agricultural and Reclamation Science/Northwest Inland Region Key Laboratory of Cotton Biology and Genetic Breeding, Ministry of Agriculture and Rural Affairs, Shihezi 832000, China; 2 College of Modern Agriculture and Food Engineering, Xinjiang Vocational University of Technology, Kashi 844004, China; 3 Tumushuke Agri-Tech R&D Center, Xinjiang Academy of Agricultural Reclamation Sciences, Tumushuke 843900, China

**Keywords:** upland cotton, fruiting branch node height, quantitative trait loci, bulked segregant analysis, SNP-based genotyping

## Abstract

The height of the first fruiting branch node (HFFBN) is a core indicator for mechanical harvesting of cotton, and the development of molecular markers for this trait is important for accelerating the breeding process. In this study, using bulked segregant analysis coupled with whole-genome sequencing (BSA-seq), one quantitative trait locus (QTL) associated with the HFFBN was mapped; a molecular marker, qFBH7, associated with the HFFBN of cotton was developed; and its application value was systematically evaluated. A total of 20 lines with extreme phenotypes were selected from the recombinant inbred lines constructed using upland cotton Z3-146 and Z3-147 as parental lines. The screened lines with extreme phenotypes were used to construct the extreme high-HFFBN pool and the extreme low-HFFBN pool, which were subsequently used for BSA-seq. Using the upland cotton genome as a reference, relevant QTLs were mapped by BSA-seq. One relevant candidate region was identified, with a total length of 2.25 Mb. The validation experiments revealed that the genotyping results of the KASP_FBH7_03 molecular marker in the parental lines Z3-146 and Z3-147 were completely consistent with the BSA-seq data: Z3-146 had the TT genotype, and Z3-147 had the CC genotype. Among the 66 samples from the natural population, there was a significant difference (*p* < 0.05) in the HFFBN between the CC and TT genotypes, and the mean HFFBN of the TT genotype was greater than that of the CC genotype. In summary, the KASP_FBH7_03 molecular marker can be effectively used for selective breeding for the HFFBN of cotton, and the TT genotype has a positive regulatory effect on the HFFBN. This study not only provides resources for breeding cotton varieties suited to mechanical harvesting but also offers a robust tool for molecular marker-assisted selection.

## 1. Introduction

Cotton is an important cash crop and raw material worldwide. With the adjustment of the layout of China’s cotton industry, Xinjiang has become China’s largest producer and exporter of high-quality cotton; thus, ensuring the safety of cotton production in Xinjiang is crucial to the development of China’s cotton industry [1,2,3]. As a key agronomic trait, the height of the first fruiting branch node (HFFBN) can indirectly affect the economic yield of cotton. The HFFBN, also known as the height of the first fruiting branch, refers to the vertical distance from the cotyledonary node to the point of the first fruiting branch [4,5]. The HFFBN is important for the structure of cotton plants and directly affects the ventilation and light conditions in the lower part of the plant and the adaptability of the plant to mechanical harvesting. Representative cotton plants are usually selected at the maturity stage to measure the height from the cotyledonary node to the position of the first fruiting branch (in cm) [6,7]. Early studies have focused mostly on the node of the first fruiting branch (NFFB), i.e., the number of nodes that result in the generation of the first fruiting branch, whereas the HFFBN comprehensively reflects the joint effect of the number of nodes and internode length [8].

The HFFBN is a quantitative trait that is controlled by multiple genes and has high heritability. Liu et al. analyzed early-maturing cotton varieties from Xinjiang and reported that the coefficient of variation (CV) of the HFFBN ranged from 14.40 to 16.91%, and the broad-sense heritability was 85.61%, indicating that the HFFBN was under strong genetic control and was suitable for selection [6]. Through correlation analysis, Shi reported that the HFFBN was significantly positively correlated with the growth period (r = 0.887) and was less affected by environmental factors; thus, the HFFBN can be used as a reliable indicator for early maturity selection [7].

Considerable progress has been made in mapping QTLs for the HFFBN using molecular markers. Li et al. [1] used two F_2_ populations to detect six QTLs highly correlated with the HFFBN, which were located on chromosomes 9, 17, and 25, and explained 5.2–13.9% of the phenotypic variation. In the F_2_ population of Jin 13 × Hai 7124, Li et al. [5]. identified QTLs that simultaneously controlled plant height and the NFFB on chromosome A3, indicating that they may have genetic linkage or pleiotropic effects. Qi et al. used a high-density genetic map to map eight QTLs for the HFFBN in the F_2:3_ population of Z571 × CCRI49, which were distributed on chromosomes 3, 4, 7, 9, and 23 and could explain 2.2–9.6% of the variation; moreover, some of these QTLs colocalized with gibberellin and cytochrome P450 synthesis-related genes, providing clues for the molecular regulatory mechanism of the HFFBN [9].

Several QTLs and related genes associated with the HFFBN have been identified in cotton; however, given the complex quantitative nature of this trait, our understanding of the functions and regulatory mechanisms of these genes remains incomplete. Further research is needed to identify the HFFBN-relevant genes to fully understand their role in regulating the HFFBN. In this study, an F_2:3_ population constructed from Z3-146 and Z3-147 and modern molecular biology techniques was used to study upland cotton; using next-generation sequencing (NGS) and bulk segregation analysis (BSA), QTLs in the upland cotton genome associated with the HFFBN were identified, and candidate genes that may play key roles in the regulation of the HFFBN were subsequently screened. Ribonucleic acid (RNA)-seq analysis was used to verify the expression levels of these candidate genes in the regulation of the HFFBN, providing important theoretical guidance and research insights for further exploration of the mechanisms regulating cotton plant height and offering a theoretical basis for breeding cotton with an ideal plant architecture.

## 2. Materials and Methods

### 2.1. Plant Materials and Phenotyping in the Field

A population of 285 F2 individuals was developed from the cross Z3-146 × Z3-147 and selfed to F2:3 for initial phenotyping and BSA-seq (using extreme individuals). Among these, 155 families segregating for HFFBN were selected and advanced to F2:5 via single-seed descent alongside the remaining 130 stable families. In 2025, 440 F2:5 entries were evaluated in Shihezi, comprising one representative line from each of the 285 original families plus 155 additional sub-lines from the segregating families for fine mapping. Plants were grown in two-row plots under a standard Xinjiang drip-irrigation system, with a narrow–wide row spacing of 10 cm and 66 cm (a ‘one film, six rows’ configuration) and a within-row plant spacing of 13 cm. In the seedling stage, the parents and each group of plants were numbered, and fresh leaves were collected, immediately frozen in liquid nitrogen, and stored at −80 °C for deoxyribonucleic acid (DNA) extraction. Standard field management practices were implemented throughout the growing season.

### 2.2. BSA-Seq

Twenty seeds were randomly selected from the hybrid population and planted in the experimental field of the Xinjiang Academy of Agricultural and Reclamation Sciences. Five plants were randomly selected from each parental and progeny population to measure the HFFBN at the boll opening stage. The 20 lines of the tallest and lowest plants were selected to construct the high plant pool (H-pool) and low plant pool (L-pool), respectively. The leaves and internode tissues were collected and rapidly frozen in liquid nitrogen, after which the genomic DNA was extracted.

#### 2.2.1. DNA Extraction, Library Construction, and Sequencing

A total of 1.5 μg of genomic DNA extracted from each extreme population using the cetyltrimethylammonium bromide (CTAB) method served as the raw material for library construction. A TruSeq Nano DNA HT Sample Preparation Kit (Illumina, San Diego, CA, USA) was used to construct the sequencing library according to the manufacturer’s protocol. Genomic DNA was fragmented to an average size of 350 bp and used to construct paired-end sequencing libraries using the NEBNext^®^ Ultra^TM^ DNA Library Prep Kit (NEB, Ipswich, MA, USA) following the manufacturer’s instructions. The libraries were sequenced on the Illumina HiSeq 4000 platform (Illumina, San Diego, CA, USA) with 150-bp paired-end reads.

#### 2.2.2. Reference Genome Alignment, Single Nucleotide Polymorphism (SNP) Detection, and Annotation

Raw sequencing data need to undergo a series of quality control procedures to remove human errors before being used for reference genome alignment and subsequent analysis. After quality control processing, the sequencing data of the samples were aligned to the reference genome of upland cotton (TM-1 ZJU v2.1) using Burrows–Wheeler Aligner (BWA v0.7.15) software [10]. The comparison results were subsequently converted to a bam file using SAMtools software [11]. The Unified Genotyper function in the Genome Analysis Toolkit (GATK v4.2.6.1) software [12] was used to detect SNPs and insertions–deletions (InDels) in each sample, and different parameters in GATK were used to filter the SNPs and InDels to avoid the influence of low-quality or invalid molecular markers on the subsequent analysis. Finally, the filtered SNPs and InDels were annotated in the reference genome file using ANNOVAR (20210601) software [13]. A detailed sample-wise quality control summary is provided in Table 1.

#### 2.2.3. Candidate Region Analysis and Gene Identification

When the upland cotton genome TM-1 was used as the reference, the number of reads in the progeny pool that were the same as or different from the reference genome at a specific base position was compared. The proportion of the number of nonidentical reads in the total number was calculated, which was defined as the SNP index. The calculation method of the InDel index was essentially the same as that of the SNP index, except that InDels involve DNA fragments of varying lengths rather than single bases. If loci with a SNP/InDel index < 0.3 appeared in the two progeny pools, they were filtered out to reduce the impact of sequencing and alignment errors [1]. A window size of 1 Mb and a step size of 1 kb were used as the standard parameters for the sliding window method, and this method was used to plot the distribution of the SNP/InDel index on the chromosome. The difference in the SNP/InDel index in the two offspring pools was calculated as Δ(SNP/InDel index). The statistical confidence intervals (CIs) required for the SNP/InDel index correlation analysis were set to 95% and 99% according to standard methods. Based on the calculated SNP/InDel index and Δ(SNP/InDel index), the candidate interval was identified as the window where the SNP/InDel index differed significantly between the two pools and where the Δ(SNP/InDel index) met a certain confidence level. Among the candidate intervals, the SNP/InDel with a SNP/InDel index of 1 in one pool and 0 in the other pool was selected as the original candidate SNP. Based on the annotation information for these SNPs, SNPs located within exons and representing significant mutations, such as non-synonymous mutations, stop-gain mutations, and stop-loss mutations, were selected as candidate SNPs, and the genes in which they are located were designated as candidate genes.

### 2.3. Development of Candidate Region Molecular Markers

Upon identifying differential SNP loci between the two parents within the candidate region, we selected those that were homozygous in both parental lines and polymorphic between them for the development of kompetitive allele-specific PCR (KASP) markers. Two specific upstream primers and one universal downstream primer were designed based on the flanking sequences of the selected SNP sites. The primers were synthesized by Sangon Biotech (Shanghai, China) Co., Ltd. Equal amounts of genomic DNA from individuals within each of the extremely resistant and susceptible bulks were pooled and used as templates. Polymorphic KASP markers, initially identified between the two parental lines, were then genotyped across these bulks and subsequently validated in a panel of natural accessions.

The PCR components consisted of KASP 2× Master Mix [Standard 6-carboxy-X-rhodamine (ROX)] containing Taq DNA polymerase, a FAM/VIC universal fluorescent probe, deoxyribonucleotide triphosphates (dNTPs), buffer, MgCl_2_, and the reference dye ROX (Standard ROX).

The phenotypic data of the HFFBN and genotypic data for the selected KASP markers from 200 samples of the F_2_ population and 200 samples of the wild population were analyzed using Excel 2021. SPSS 25.0 software was used for statistical data analysis, and a *t*-test was used to detect the differences between the markers and phenotypes of different genotypes (*p* < 0.05 indicates that the difference was statistically significant, *p* < 0.01 indicates that the difference was highly significant, and *p* < 0.001 indicates that the difference was extremely statistically significant). The strength of the association between each marker and the phenotypic trait was analyzed to verify the practicality of the HFFBN molecular markers and establish the phenotype–genotype relationship.

## 3. Results

### 3.1. Statistical Analysis of First Branch Height (FBH)

**Figure 1:**
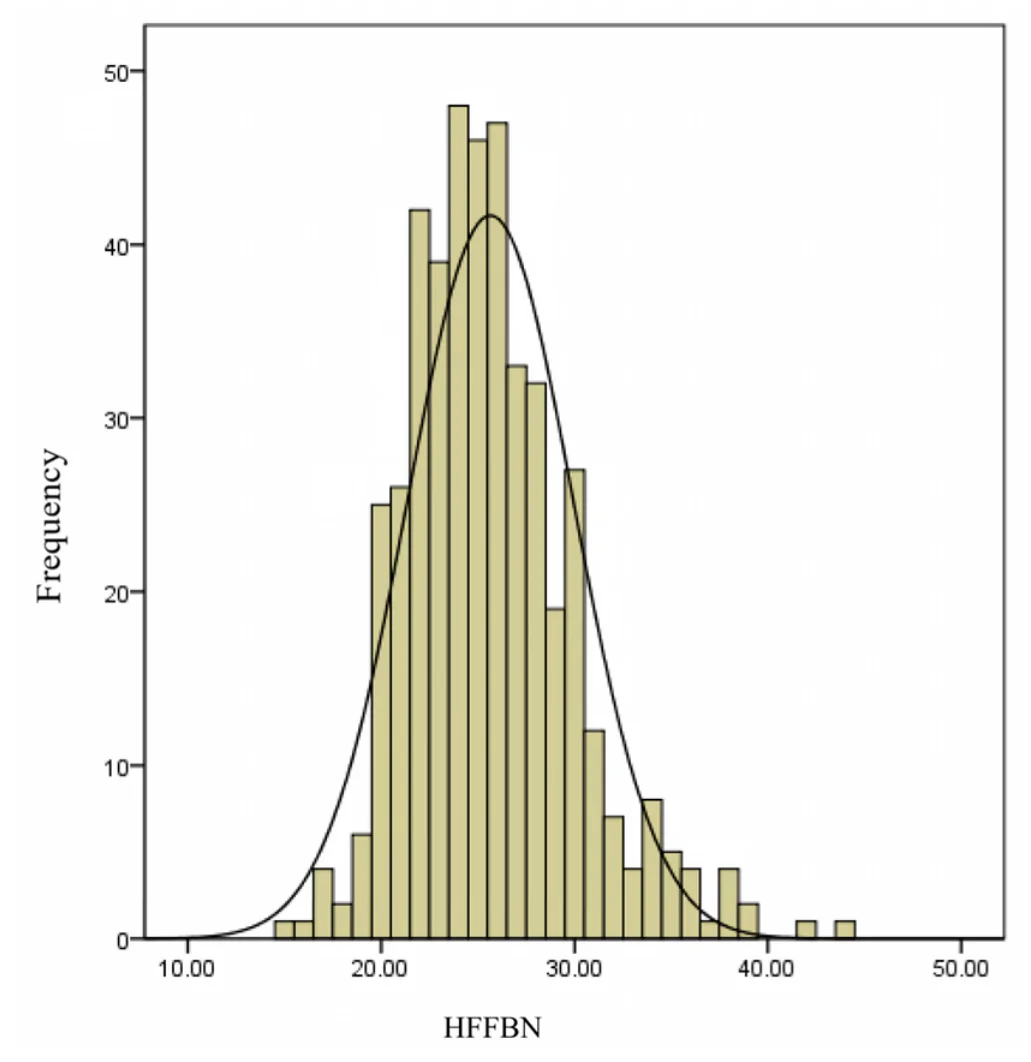
High-frequency distribution of the HFFBN in population F_2_.

Significant differences were detected in the HFFBN between the parental lines (Table 2). After harvest, IBM SPSS Statistics 19.0 was used to plot the high-frequency distribution map of the HFFBN of the population, and a normal distribution test was performed. The results revealed that both the skewness and kurtosis were less than 1, indicating that the HFFBN of the population followed a normal distribution. Therefore, we speculated that the HFFBN of cotton in this population was a quantitative trait controlled by major genes (Table 3, Figure 1). Afterward, the HFFBN was sorted from low to high in Excel, and 20 plants with high HFFBN and 20 plants with low HFFBN were selected to form two extreme pools related to the HFFBN.

### 3.2. BSA-Seq Quality Assessment

The two parental lines and the two extreme mixed pools were sequenced on the Illumina NovaSeq sequencing platform using the PE150 sequencing strategy, and a total of 962,710,908 bp of raw data were obtained. Through quality control measures such as the removal of linkers and low-quality reads, a total of 704,218,746 clean bases were obtained. Quality assessment revealed that the GC content of these reads ranged from 35.35–35.66%, and the quality of the sequencing data was high (Q20 ≥ 96.39%, Q30 ≥ 90.81%), indicating that these reads could be used for further analysis.

### 3.3. Mapping Analysis, SNP Detection, and Annotation

**Figure 2:**
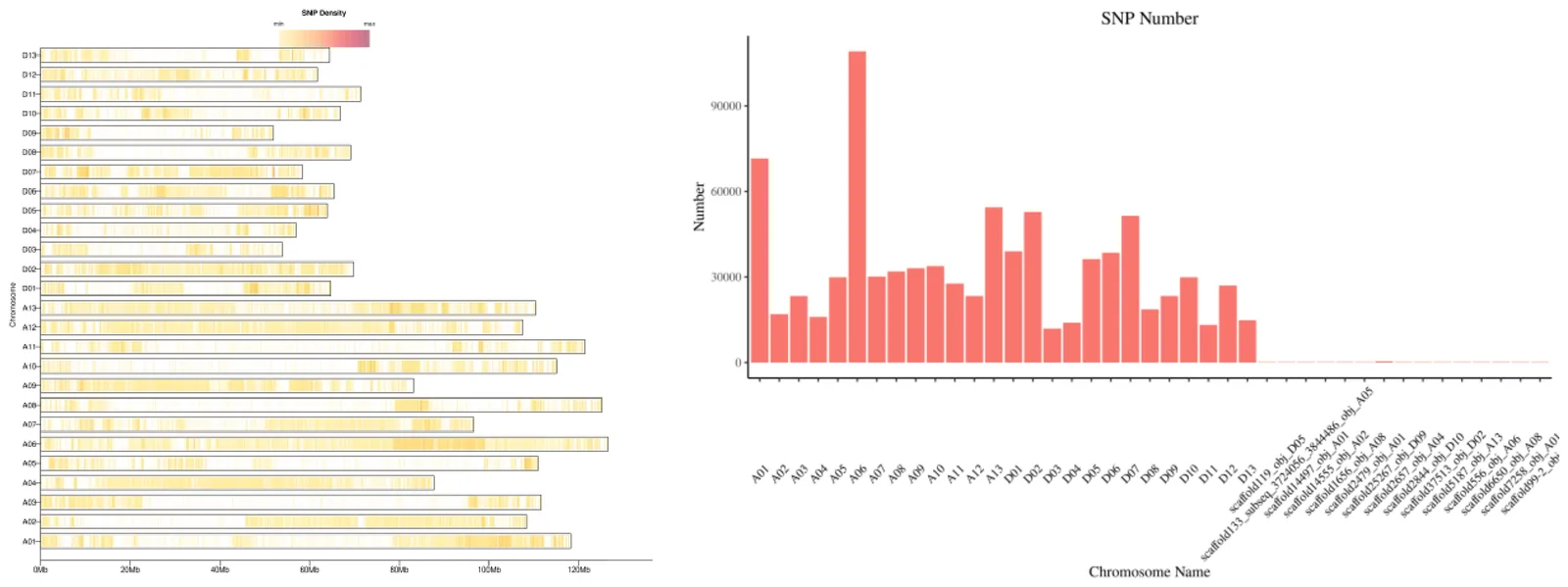
Distribution and statistics of chromosomal SNPs.

Using the TM-1 genome in the integrated database as the reference genome, a total of 1,907,124,610 reads were obtained after the quality control data were mapped. The mapping rate of the samples ranged from 99.50 to 99.66%; the average sequencing depth of the parental lines was 20×, and the average sequencing depth of the mixed pool was 30×, indicating that the reference genome had uniform and random coverage and a high mapping rate, which was conducive to subsequent SNP screening and labeling (Figure 2).

After screening, a total of 13,202 biallelic SNPs and 2336 biallelic short insertions or deletions (InDels) were identified.

### 3.4. Candidate Region Localization and Gene Screening

**Figure 3:**
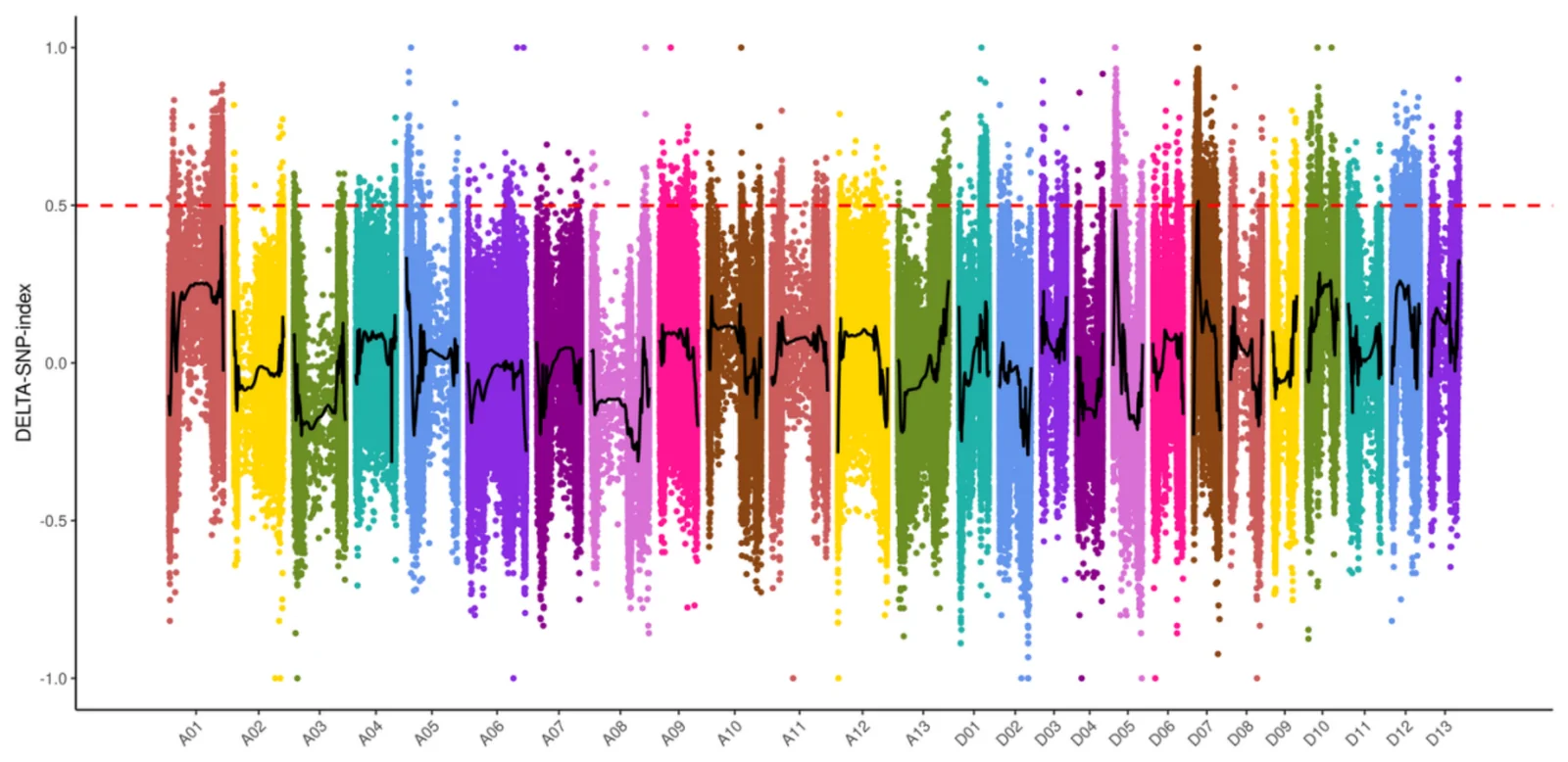
Distribution of SNP-index association values on chromosomes. SNPindexA: The distribution of SNP-index values of the HC1 samples on the chromosomes. SNPindexB: Distribution of the SNP-index values of the HC2 samples on the chromosomes. Delta-SNP-index: Distribution of ∆ (SNP-index) values on the chromosomes; the red line represents the 95% CI.

The distribution of the SNP index on the chromosome was plotted for visualization (Figure 3). Afterward, the difference in the SNP-index values of the two offspring pools was calculated to obtain ∆ (SNP-index) = SNP-index (extreme trait A) − SNP-index (extreme trait B). Following 10,000 permutation tests, a 99% genome-wide confidence threshold was established for ∆ (SNP-index). Applying this threshold, only one genomic region—an interval on chromosome D07—exceeded the significance cutoff (Figure 3). Subsequent analysis of non-synonymous mutations within the exons of this candidate region identified 153 annotated genes, ultimately narrowing the candidate locus to a physical interval spanning bases 8,379,614–10,628,581 on D07. These 153 genes, all of which carry non-synonymous SNP variants in their exonic regions, were then subjected to Gene Ontology (GO) and Kyoto Encyclopedia of Genes and Genomes (KEGG) pathway enrichment analyses to explore their potential biological functions.

### 3.5. Gene Ontology (GO) Classification and Enrichment Analysis

**Figure 4:**
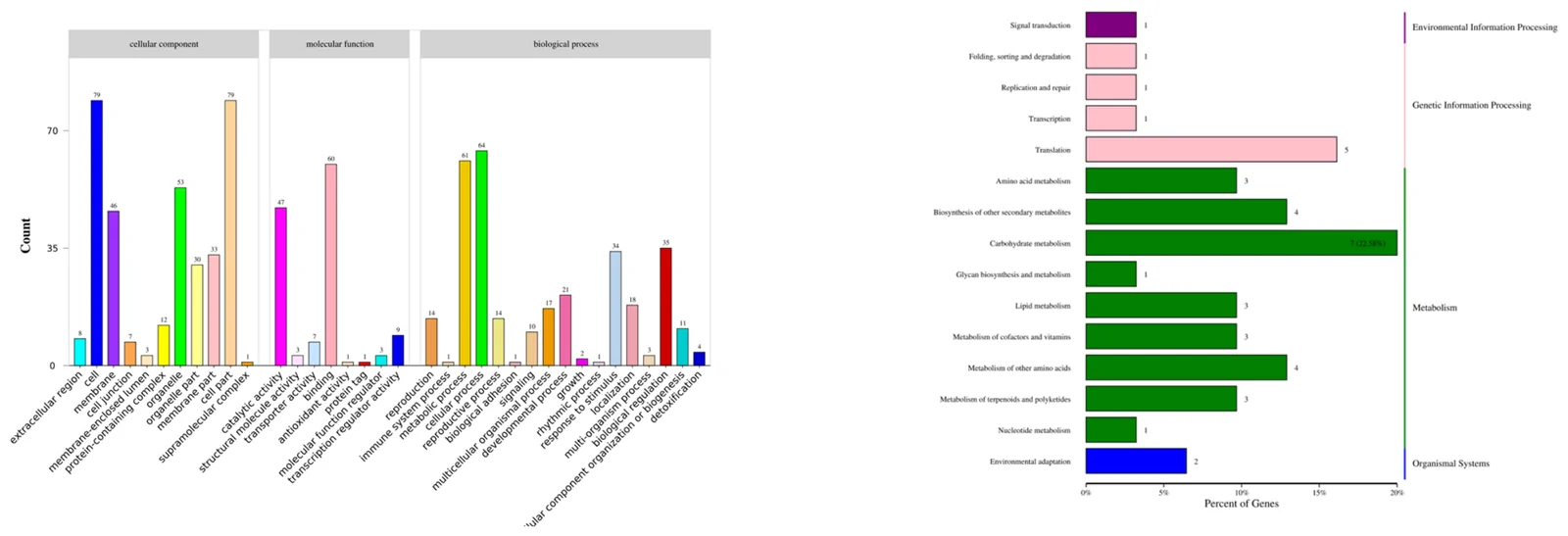
GO enrichment and KEGG pathway annotation of the candidate genes.

GO annotation of the mutated genes in the candidate region was performed using InterProScan software (v5.53). The genes were subjected to GO enrichment analysis and classified according to biological process (BP), molecular function (MF), and cellular component (CC). The genes were annotated into 2 functional groups (Figure 4), including 5 BPs and 6 MFs. In terms of BP, these genes were primarily involved in cellular protein modification, carbohydrate metabolism, and stress responses. In terms of MF, these genes were primarily involved in molecular functions, ion binding, kinase activity, and other functions. These findings suggest that the developmental process of the HFFBN may be related to cellular protein modification, carbohydrate metabolism, and biological and catabolic processes. Moreover, ion binding and kinase activity may be involved in the development of the HFFBN.

To identify the metabolic pathways involved in gene enrichment, pathway enrichment analysis of the candidate genes was performed using the Kyoto Encyclopedia of Genes and Genomes (KEGG) pathway database. Genes were mainly enriched in three protein families: plant hormone signal transduction, starch and sucrose metabolism, and the phenylalanine biosynthesis pathway (Figure 4). These findings indicate that plant hormone signal transduction, starch and sucrose metabolism, and phenylpropanoid biosynthesis play important roles in the development of the HFFBN.

The results of the KEGG enrichment analysis were essentially consistent with those of the GO enrichment analysis, further indicating that biosynthetic and metabolic pathways play important roles in the development of the HFFBN of cotton.

### 3.6. Fine Mapping of Candidate Genes and Development of Molecular Markers

**Figure 5:**
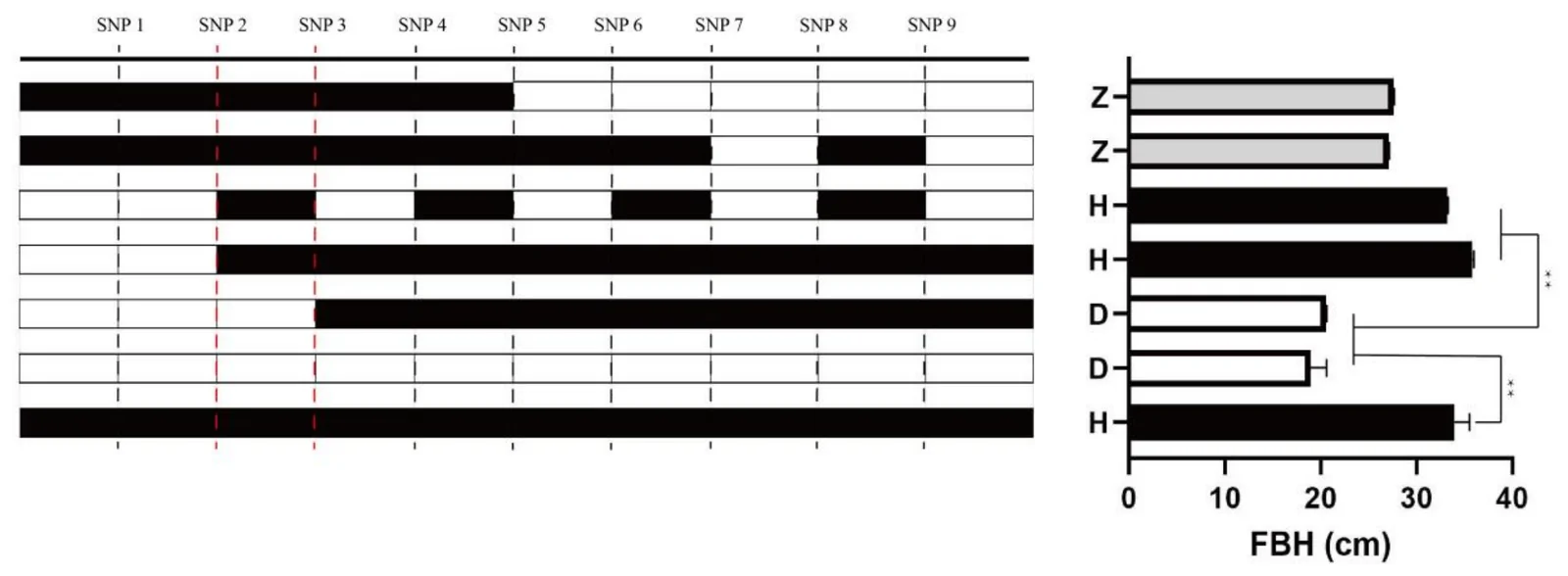
Fine mapping of qFBH7 using a hybrid population of Z3-146 and Z3-147. ** indicate significant differences at *p* < 0.05. (Black represents materials with high FBH values, white represents materials with low FBH values, and gray represents hybrid type materials).

The F_2_ generation after selfing was used for genotyping analysis, and the corresponding F_2:3_ plants were used for phenotypic analysis of the HFFBN to narrow the QTL interval of qFBH7. A total of 100 recombinants were obtained, of which 36 lines had a higher HFFBN (FBH_H) and 17 lines had a lower HFFBN (FBH_L). Using 100 individuals from three generations of inbred populations and 10 SNP variant markers, qFBH7 was ultimately mapped within a 595.493 kb interval (between SNP2 and SNP3). According to the annotation results, there were a total of 48 genes in this interval (Figure 5).

**Figure 6:**
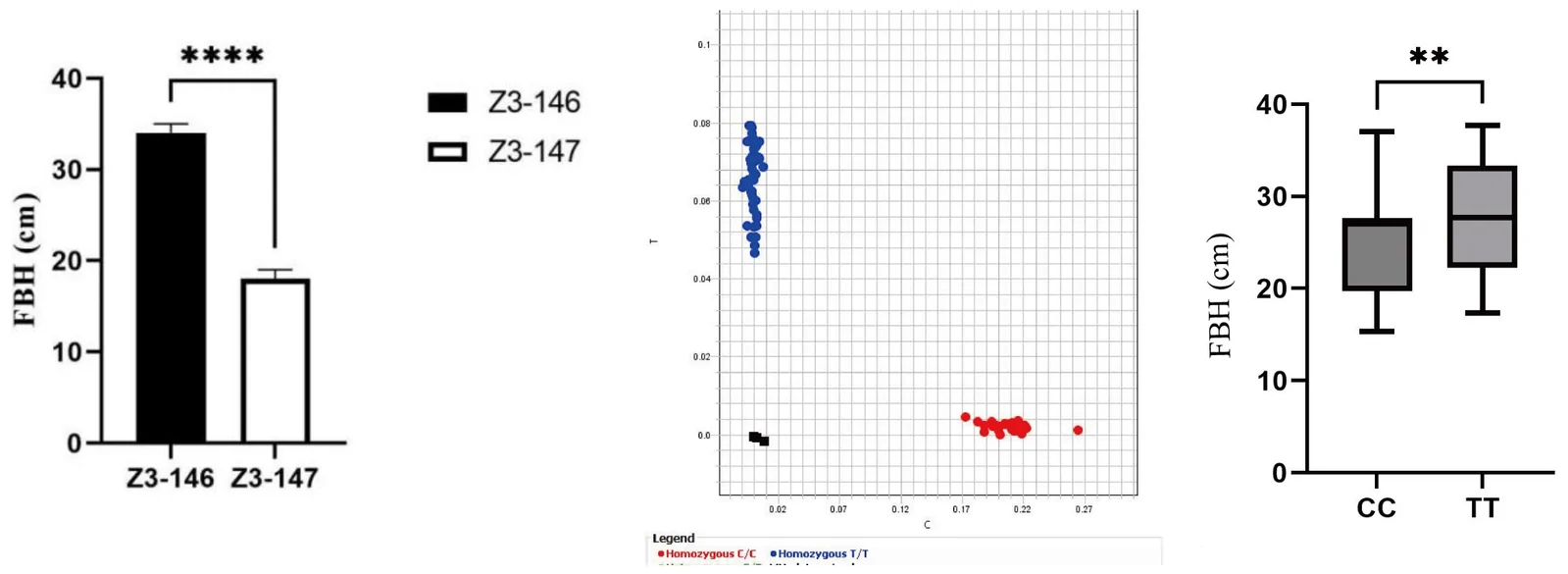
Analysis of the differences in the HFFBN trait between the two parental lines, genotyping scatter plot for the development of the KASP marker KASP_FBH7_03, and analysis of the phenotypic differences between the two genotypes, **** and ** indicates a highly significant difference.

**Figure 7:**
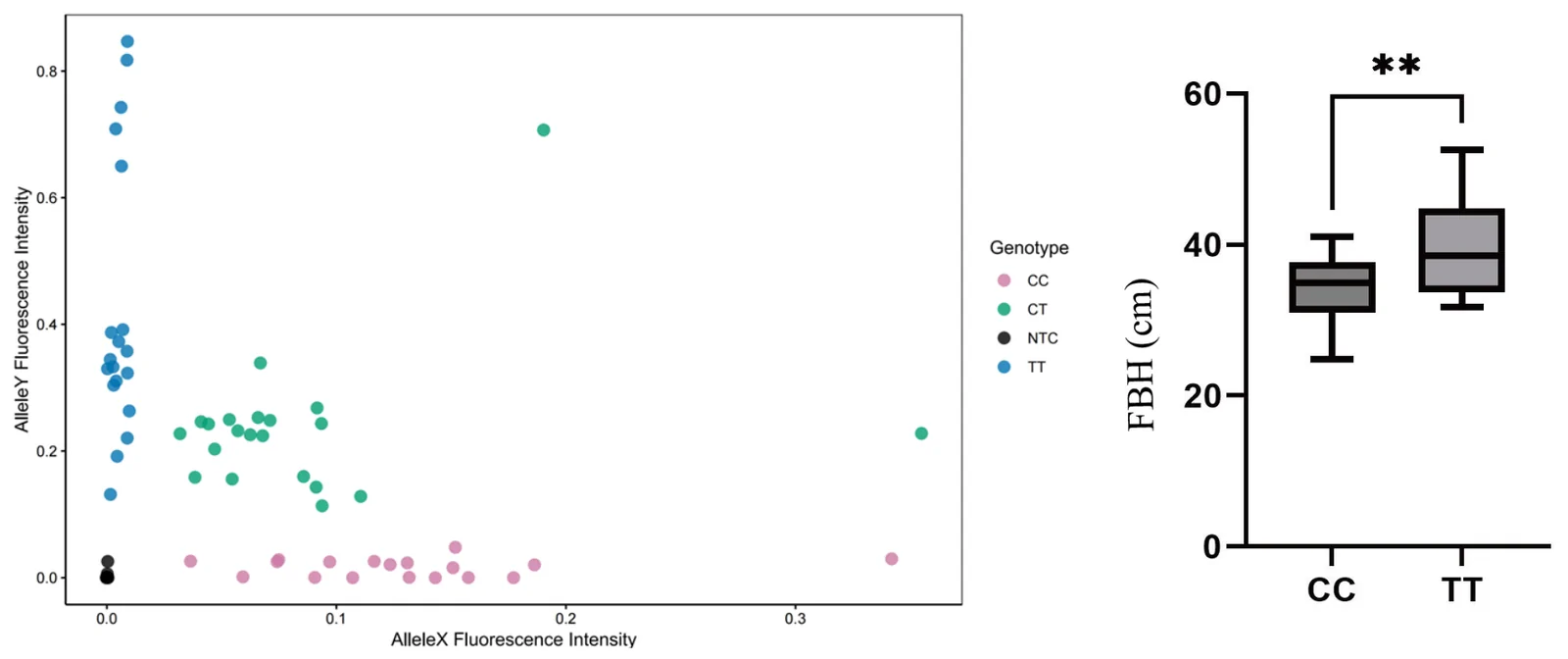
Scatter plot of natural population data combining the HFFBN phenotype with the KASP marker genotype; one-way analysis of variance (ANOVA) was performed; ** indicates a highly significant difference.

Furthermore, through the analysis of the two markers, a KASP marker was developed. At the qFBH7_03 marker locus, the two parental lines, Z3-146 and Z3-147, exhibited the T and C genotypes, respectively (Figure 6). Additionally, there was a highly significant difference (*p* < 0.001, ***) between the two parental lines in terms of the HFFBN phenotype. KASP genotyping was performed on 100 randomly selected DNA samples from the F_2:3_ generation. The results revealed that this KASP marker (KASP_FBH7_03) was very useful for genotyping the F_2_ population, and its genotypes and phenotypes were highly consistent (Figure 6). For KASP_FBH7_03, the TT, CC, and CT genotypes corresponded to the high-HFFBN, low-HFFBN, and heterozygous groups, respectively (Figure 6). A *t*-test revealed that there was a significant difference in the HFFBN between the high-HFFBN and low-HFFBN groups (*p* = 0.001) (Figure 6). At the same time, 66 materials were randomly selected from the natural population, and the genotyping of these 66 materials at this locus was performed. The results revealed that all the materials could be divided into three types (including the T/C heterozygous type). Data analysis of the HFFBN phenotype of these 66 materials from the natural population revealed that there was an extreme difference between the HFFBN phenotype of the two genotypes (Figure 7).

## 4. Discussion

### 4.1. Parental Selection, Population Construction, and Analysis of the HFFBN

Biparental hybrid populations are ideal materials for the detection of QTLs. Biparental hybrid populations are bred through crossing, selfing, and molecular marker-assisted selection. These populations contain only genetic contributions from the two donor parents and have a clear genetic background, which greatly minimizes the confounding interference caused by complex genetic backgrounds. As research has progressed, SNPs and InDels, as types of genetic variation, have gradually become the markers of choice for BSA-seq, and their application in BSA-seq has been progressively validated. For example, regions associated with disease resistance have been preliminarily mapped in tomato (*Solanum lycopersicum* L.) [13] and pigeon pea (*Cajanus cajan* L.) [14] through association analysis of InDels. In addition, Toporek mapped the downy mildew resistance candidate interval to chromosome 9 using the simple sequence repeat (SSR) marker developed from the recombinant inbred line (RIL) population of MR-1 × A-nanas Yokneam [15]. Our results demonstrate that KASP_FBH7_03 is a diagnostic marker for HFFBN, with validation across natural populations confirming its reliability in distinguishing high- and low-performance genotypes. This marker provides an immediate, practical tool for molecular-assisted breeding, enabling early-generation selection without compromising breeding cycle efficiency. Additionally, the observed allelic variation between different germplasm groups lays a foundation for future exploration of the domestication history of this locus, although further fine-mapping and functional studies are needed to dissect the underlying causal variant. These results lay a preliminary foundation for further dissecting the genetic basis of HFFBN and may facilitate the development of molecular tools for breeding cotton varieties better adapted to mechanical harvesting.

*Gossypium hirsutum* and *Gossypium barbadense* share a common origin but have undergone independent evolution, resulting in significant divergence in their genomes and marked differences in plant morphology, yield, and fiber quality [16,17]. In this study, the F2:3 population, generated from the crossing of upland cotton Z3-146 and Z3-147, exhibited extensive variation in the HFFBN, and the frequency distribution of the phenotypic values exhibited a bell-shaped pattern with a single peak, suggesting an approximately symmetrical distribution (Figure 1). The F_2_ hybrid population represents a permanently segregating population, in which the variation sites are largely homozygous, conferring a relatively stable genetic composition and high heritability [18]. These populations with a clear genetic background are particularly suitable for QTL mapping analysis because the results of linkage analysis are less affected by population structure. Direct measurement of genotypes and phenotypes in the subsequent generation can reveal the chromosomal locations and genetic effects of genes that control the HFFBN through genotype–phenotype correlations. In many crop species, the application value of the F_2_ hybrid population in improving the accuracy of QTL analysis has been confirmed.

### 4.2. Analysis of BSA-Seq Mapping Results

In this study, BSA-seq identified two candidate regions associated with the high fruiting branch node (HFFBN) trait in cotton, with one positive regulatory site mapped to chromosome D07 (Figure 3). Given that HFFBN and plant height are two interdependent components of cotton plant architecture, it is critical to assess whether the D07-associated locus exerts a direct effect on HFFBN or indirectly influences it through modulation of plant height.

Previous studies have documented complex genetic and phenotypic relationships between these two traits. For instance, Shi et al. found a non-significant simple correlation (r = 0.0153) but a highly significant partial correlation (r = 0.8244) after eliminating other trait interferences, suggesting that their intrinsic genetic linkage can be masked by environmental or developmental noise [4]. In support of this, Li et al. (2010) identified co-localized QTLs for both traits on chromosome A3 in an F2 population (r = 0.472 **), providing direct evidence for their shared genetic basis [5].

However, the genetic correlation between HFFBN and plant height is not static. Environmental stresses, such as water deficit or defoliation, tend to affect plant height more profoundly while leaving node development relatively stable [19]. This differential sensitivity implies that the correlation could weaken under adverse conditions, as further supported by Yu et al., who reported a moderate genetic correlation coefficient (0.46, *p* < 0.01) but a negligible environmental correlation, confirming that their association is predominantly under genetic control [20].

Importantly, our BSA-seq data offer novel insights into this relationship. The candidate region on D07 identified in this study does not coincide with any previously reported plant-height QTLs (e.g., those on A3 [2]), and the phenotypic evaluation of our mapping population showed that the D07-associated KASP markers were significantly associated with HFFBN (*p* < 0.001). This suggests that the D07 locus represents an HFFBN-specific regulatory element, rather than a pleiotropic modulator of overall plant stature. Therefore, while breeders should remain cognizant of the general positive correlation between HFFBN and plant height, the markers developed from this D07 region may serve as a more direct and reliable tool for selecting ideal plant architecture without confounding effects on height.

### 4.3. Development of HFFBN Markers

The SNP markers developed in this study were designed and validated following the KASP marker development strategy established in previous studies [21,22] and provide a practical tool for marker-assisted selection targeting HFFBN in cotton. BSA-seq has proven to be a powerful and cost-effective approach for rapid QTL mapping and marker development in crops such as wheat, where it has been successfully applied to dissect loci controlling disease resistance and kernel size [23,24]. However, the genetic architecture of plant architecture traits, particularly the high fruiting branch node (HFFBN) in cotton, remains less explored despite its critical importance for mechanical harvesting. Our previous study identified a major locus associated with HFFBN on chromosome D07. To translate this positional information into practically applicable markers, we designed 9 SNP markers within the candidate interval. Ultimately, the KASP FBH7_03 marker classified all natural materials into three distinct genotypic categories (homozygous T, homozygous C, and heterozygous T/C), demonstrating its high discriminatory ability and robustness across different genetic backgrounds (Figure 6).

Notably, this single marker effectively differentiated the accessions into two distinct phenotypic groups: lines carrying the T allele exhibited significantly higher HFFBN values than those carrying the C allele (*p* < 0.01, *t*-test; Figure 6 and Figure 7), demonstrating its potential as a reliable indicator for high-HFFBN selection. This finding is particularly relevant for cotton breeding, as it implies that simply selecting for the favorable homozygous genotype may not be optimal; instead, marker-assisted selection could leverage the heterozygote advantage. Collectively, these SNP markers not only refine the resolution of the D07 locus but also provide a ready-to-use toolkit for breeders aiming to optimize plant architecture without the need for time-consuming phenotypic screening.

## 5. Conclusions

The F_1_ generation was constructed using Z3-146 and Z3-147 as parents. An F_2_ segregating population was formed by F_1_-generation selfing. From the F_2_ segregating population, 20 plants with extreme HFFBN were selected to construct the extreme mixed pools, and BSA-seq was performed. One QTL associated with the HFFBN trait was mapped on chromosome D07. Within this candidate interval, one pair of KASP molecular markers (KASP-qFBH7) tightly linked to the HFFBN was developed. In the F_2_ generation segregating population, the proposed marker was used to classify the offspring into two groups, namely high-HFFBN and low-HFFBN, and further classify the material from the natural population into these two groups. Therefore, the molecular marker developed at this SNP locus can be used for breeding cotton varieties with HFFBN values suited to mechanical harvesting.

## Figures and Tables

**Table 1:** Summary of sequencing data quality and alignment statistics.

SampleID	Raw_Reads	Clean_Bases	Q20(%)	Q30(%)	Mapping Rate (%)
Z3-146	212,594,782	63,965,505,016	98.58	95.83	99.63
Z3-147	215,983,207	64,942,993,248	98.53	95.69	99.56
High-pool	278,555,781	83,765,563,028	98.46	95.5	99.57
Low-pool	255,577,138	76,846,695,010	98.46	95.51	99.56

Q20/Q30, percentage of bases with Phred quality score > 20 or 30. Mapping rate, percentage of clean reads uniquely aligned to the reference genome.

**Table 2:** Phenotypic values of the HFFBN of the parents.

Parents	HFFBN/cm	HFFBN/cm	Average
Z3-146	35 a	37 a	34 a
Z3-147	16 b	15 b	18 b

Different lowercase letters indicate significant differences at *p* < 0.05, while the same letters indicate no significant difference.

**Table 3:** High-frequency distribution of the HFFBN in population F_2_.

	Mean	Standard Deviation	Coefficient of Variation	Skewness	Kurtosis	Maximum Value	Minimum Value
HFFBN (cm)	25.64	4.27	3.98	0.83	1.31	44.0	15.0

## Data Availability

The raw data supporting the conclusions of this article will be made available by the authors on request.
